# Viable SARS-CoV-2 Omicron sub-variants isolated from autopsy tissues

**DOI:** 10.3389/fmicb.2023.1192832

**Published:** 2023-05-22

**Authors:** Santiago Maffia-Bizzozero, Cintia Cevallos, Federico Remes Lenicov, Rosa Nicole Freiberger, Cinthya Alicia Marcela Lopez, Alex Guano Toaquiza, Franco Sviercz, Patricio Jarmoluk, Cristina Bustos, Adriana Claudia D’Addario, Jorge Quarleri, M. Victoria Delpino

**Affiliations:** ^1^Morgue Judicial de la Nación, Buenos Aires, Argentina; ^2^Instituto de Investigaciones Biomédicas en Retrovirus y Sida (INBIRS), Universidad de Buenos Aires, Consejo Nacional de Investigaciones Científicas y Técnicas (CONICET), Buenos Aires, Argentina

**Keywords:** SARS-CoV-2, virus isolation, postmortem tissue, Vero E6 cell line, omicron variants

## Abstract

**Introduction:**

Pulmonary and extrapulmonary manifestations have been described after infection with SARS-CoV-2, the causative agent of coronavirus disease 2019 (COVID-19). The virus is known to persist in multiple organs due to its tropism for several tissues. However, previous reports were unable to provide definitive information about whether the virus is viable and transmissible. It has been hypothesized that the persisting reservoirs of SARS-CoV-2 in tissues could be one of the multiple potentially overlapping causes of long COVID.

**Methods:**

In the present study, we investigated autopsy materials obtained from 21 cadaveric donors with documented first infection or reinfection at the time of death. The cases studied included recipients of different formulations of COVID-19 vaccines. The aim was to find the presence of SARS-CoV-2 in the lungs, heart, liver, kidneys, and intestines. We used two technical approaches: the detection and quantification of viral genomic RNA using RT-qPCR, and virus infectivity using permissive *in vitro* Vero E6 culture.

**Results:**

All tissues analyzed showed the presence of SARS-CoV-2 genomic RNA but at dissimilar levels ranging from 1.01 × 10^2^ copies/mL to 1.14 × 10^8^ copies/mL, even among those cases who had been COVID-19 vaccinated. Importantly, different amounts of replication-competent virus were detected in the culture media from the studied tissues. The highest viral load were measured in the lung (≈1.4 × 10^6^ copies/mL) and heart (≈1.9 × 10^6^ copies/mL) samples. Additionally, based on partial Spike gene sequences, SARS-CoV-2 characterization revealed the presence of multiple Omicron sub-variants exhibiting a high level of nucleotide and amino acid identity among them.

**Discussion:**

These findings highlight that SARS-CoV-2 can spread to multiple tissue locations such as the lungs, heart, liver, kidneys, and intestines, both after primary infection and after reinfections with the Omicron variant, contributing to extending knowledge about the pathogenesis of acute infection and understanding the sequelae of clinical manifestations that are observed during post-acute COVID-19.

## 1. Introduction

SARS-CoV-2 has caused millions of deaths worldwide. Pulmonary and extrapulmonary manifestations have been described after infection with SARS-CoV-2, the causative agent of COVID-19 (Elrobaa and New, 2021). Until now, most efforts have been made to understand acute COVID-19 in the early stages. Most COVID-19 patients recover within a few days to a few weeks after infection. However, people who have been exposed to SARS-CoV-2 may have a variety of new, recurring, or ongoing health sequelae. These disorders are known as post-acute COVID-19, also known as long-COVID, that cannot be explained by other diagnoses and take at least 4 weeks after infection to appear. Post-acute COVID-19 symptoms can affect anyone who contracted the infection, but some groups might be more at risk than others for developing such sequelae, such as those who have experienced more severe COVID-19 illness, especially those who were hospitalized or needed intensive care (Nalbandian et al., 2021; Sanyaolu et al., 2022; Soriano et al., 2022). Nevertheless, post-acute COVID-19 has been observed even among young and middle-aged adults after six to 12 months after mild infection (Peter et al., 2022). Some studies have reported that COVID-19 vaccines had a protective effect against long-COVID even among patients vaccinated before or after SARS-CoV-2 infection/COVID-19 (Al-Aly et al., 2022; Gao et al., 2022; Nascimento et al., 2023). Additionally, the evidence shows that a SARS-CoV-2 reinfection further increases risks of death, hospitalization, and sequelae in multiple organ systems in the acute and post-acute phase, particularly in those who were unvaccinated and had one vaccination or two or more vaccinations before reinfection (Bowe et al., 2022). The underlying mechanisms of post-acute COVID-19 pathogenesis are not well understood, but an association with SARS-CoV-2 persistence in some tissues has been proposed, particularly those with high expression of the viral receptor ACE2, thus acting as a viral reservoir for SARS-CoV-2 (Neurath et al., 2021; Chen et al., 2022). Several reports have described the persistence of viral RNA and/or antigens in gastrointestinal tissues, cardiovascular system, reproductive tissues, brain, muscles, breast tissue, lymph nodes, stool, and urine (Lamers et al., 2020; Trypsteen et al., 2020; Martin-Cardona et al., 2021; Neurath et al., 2021; Chen et al., 2022; Cheung et al., 2022; Goh et al., 2022; Stein et al., 2022; Tejerina et al., 2022). Fundamentally, the underlying significance of established risk factors, including age, obesity, and diabetes, as well as why some people advance to severe forms of COVID-19 after a two- to three-week period, is still unknown. Only autopsy investigations can provide an answer to disease pathophysiology and death queries (Layne et al., 2022). These autopsy studies have provided evidence of the ability of SARS-CoV-2 to infect multiple organs (Edler et al., 2020; Deinhardt-Emmer et al., 2021; Fitzek et al., 2021; Maiese et al., 2021). Moreover, autopsy material from different tissues revealed the persistence of SARS-CoV-2 genomic RNA for a time ranging from 22 to 27 days between death and autopsy (Stein et al., 2022). Although SARS-CoV-2 genomic RNA and even subgenomic mRNA have been found in several organs, this does not necessarily indicate that the virus can replicate and be infectious (Casagrande et al., 2020; Wolfel et al., 2020; Aquila et al., 2021; Beltempo et al., 2021; Sablone et al., 2021). Currently, host (infection time, SARSCoV-2 infection-induced and/or vaccine-induced immunity) and virus-related (variant, viral load) factors can modify the course of COVID-19 and even the probability of post-COVID occurrence. Understanding the mechanism of acute COVID-19 as well as its long-term consequences is crucial because it causes multi-organ damage (including the lungs, heart, liver, kidney, and intestine). However, comparative research between the many implicated organs is still scarce. The objectives of this study are to describe the involvement of the aforementioned organs after viral spreading, assess the SARS-CoV-2 viral load and genomic characterization, and elucidate the SARS-CoV-2 infectivity between decedents with first infection and those with SARS-CoV-2 reinfection and clinical records of COVID sequelae.

## 2. Materials and methods

### 2.1. Patients and specimen collection

This study involved 21 decedents with antemortem SARS-CoV-2 infection (named from “A” to “U”). They were subjected to minimally invasive forensic autopsy between 5 January and 6 August 2022 at the Judicial Morgue of Argentina (Table 1). Image-guided tissue sampling was performed in the right lung, right kidney, small intestine, right lobe of the liver, and left heart ventricle in regions with edema, congestive hemorrhage, and organ consolidation in the case of the right lung.

**Table 1:** Clinicopathological data from cadaveric donors.

Case	Age	Gender	SARS-CoV-2 vaccination	Vaccine	Doses	Spike-nucleotide sequences (GenBank accession numbers)	SARS-CoV-2 diagnosis (days)	Previous SARS-CoV-2 infection (months)	Autopsy records	Thoracic radiography	Pre-existing conditions	
A	0.25	F	No			OQ658550	1		Pulmonary congestion and edema	Unilateral diffuse radiopacity	No	H
B	17	F	No			OQ658553; OQ658554; OQ658555	8		Pulmonary congestion, ischemic dilated hypertrophic cardiomyopathy	Diffuse radiopacity	Undetermined
C	35	F	Incomplete	S	1	OQ658551	2		Pulmonary edema	Diffuse radiopacity	No
D	54	M	Incomplete	AZ	1	OQ658552; OQ658549	22		Lung congestion. Renal hypoplasia	Unilateral heterogeneus radiopacity	Diabetes- artherial hypertension- chronic renal insufficiency- transplant
E	59	M	No			NA	17	8	Pulmonary edema	Diffuse radiopacity	Previous SARS-CoV-2 infection-HIV
F	69	M	No			NA	10	8	General congestion. Pleural effusion.	Heterogeneous radiopacity	Previous SARS-CoV-2 infection- Chronic obstructive pulmonary disease	HN
G	71	F	Complete	SV-SV-M	3	OQ658545; OQ658556	6		Lung congestion.	Heterogeneous radiopacity	Chronic obstructive pulmonary disease- diabetes- acute myocardial infarction
H	79	M	Incomplete	SV	1	OQ658544	8		Hypertrophic and dilated heart disease. Visceral congestion	Diffuse radiopacity	Aortic stenosis
I	81	F	Complete	AZ	4	OQ677000; OQ677002	12		Pulmonary edema	Diffuse radiopacity	No
J	92	M	Complete	S-S-AZ	3	OQ676997; OQ676998	4		Congestion and pulmonary edema. Heart disease	Diffuse radiopacity	No	
K	27	M	Complete	S-S-C	3	OQ676995	14	12	Cardiac hypertrophy	Diffuse radiopacity	Previous SARS-CoV-2 infection
L	29	F	Incomplete	S	1	OQ658543	10		Cardiac hypertrophy	Diffuse radiopacity	No
M	34	F	Complete	S-M-M	3	OQ676999; OQ677001; OQ677003	23		Pulmonary edema and hemorrhage. Hypertrophic heart disease	Heterogeneous radiopacities with consolidations	No
N	50	F	No			NA	4		Hypertrophic and dilated heart disease. Hypertrophic heart disease	Heterogeneous radiopacity	Diabetes- artherial hypertension- Renal insufficiency
O	64	M	Incomplete	AZ	2	OQ658547	12		General congestion	Heterogeneous radiopacity	No
P	66	M	Incomplete	S	2	NA	28		Pulmonary edema and hemorrhage	Heterogeneous radiopacity	No
Q	69	F	No			OQ658557	6		Widespread congestion. Pleural effusion	Heterogeneous radiopacity	No
R	71	M	No			OQ658541; OQ658542; OQ676994; OQ658546	7		Hypertrophic and dilated heart disease. Visceral congestion	Heterogeneous radiopacity	No
S	84	F	Unknown			OQ658548	1		General congestion. Pleural effusion.	Diffuse radiopacity	No
T	92	M	Complete	S-S-AZ-AZ-AZ	5	OQ676996	12	6	Pulmonary edema	Heterogeneous radiopacy with consolidations	Previous SARS-CoV-2 infection
U	94	F	Incomplete	CS-AZ	2	NA	8	12	Pulmonary congestion and edema. Hypertrophic heart disease	Heterogeneous radiopacity with consolidations	Previous SARS-CoV-2 infection

AZ, AstraZeneca; CS, Covishield; S, Sinopharm; C, Cansino; SV, Sputnik V; H, Hospitalized; NH, Non-Hospitalized.

Samples were collected by forensic medical personnel when a cadaver was admitted, following international recommendations for sample collection in the context of the COVID-19 pandemic regarding the use of personal protective equipment (Centers for Disease Control and Prevention, 2020). This involved wearing a surgical jacket and trousers, goggles, an N95/FFP3 face mask, and high rubber boots, along with disposable equipment such as an impermeable surgical gown, a surgical cap, and gloves. To prevent contamination, samples were taken from cadavers in suspicious cases in an isolated environment that included restricted access to the facilities, closed rooms with negative air pressure, and appropriate decontamination and waste inactivation (Centers for Disease Control and Prevention, 2020).

The decedents ranged in age from 3 months to 94 years (mean: 61; median: 67). Eleven cadavers were female and nine were male. Small tissue samples were obtained from the lung, heart, liver, and kidney of each cadaver, as well as 16 from the small intestine, and stored in RNAlater^®^ (Sigma-Aldrich) at −80°C until use. On average, tissue samples were collected 1.9 days postmortem while the corpses were maintained at 4°C.

SARS-CoV-2 RNA was posthumously confirmed in all cases by routinely performing real-time reverse transcription-polymerase chain reaction (RT-qPCR) on nasopharyngeal swab samples. Ten of the deaths occurred after hospitalization (H) while the remaining 11 were non-hospitalized (NH). COVID-19 vaccine varied among the 13 cases representing different formulations available. Seven cases had not been vaccinated and no records were obtained for the remaining one. As a rule, nasopharyngeal swabs and radiological examinations were performed on all suspected cases to distinguish between cases where SARS-CoV-2 infection was the sole cause of death and those where COVID-19 was merely a contributing factor. However, the specific chain of events leading to their deaths is only available in clinical records that we were unable to access due to Argentine legal regulations, limiting the scope of this study.

Pre-autopsy nasopharyngeal swabs can reveal whether a corpse is infected or not. In the initial hours following death, thoracic radiological examinations (RE) and macroscopic autopsy records can provide insights into the extent and severity of the illness.

Five cases (E, F, K, T, U) were categorized as SARS-CoV-2 reinfections because SARS-CoV-2 was diagnosed for them in the past 6–12 months. This time frame reduces the probability that the positive post-mortem test was related to the first infection (Table 1). A wide range of symptoms, including fever, exhaustion, headache, shortness of breath, coughing, chest discomfort, heart palpitation, digestive issues (diarrhea, stomach pain), or neurological issues (anxiety, disturbed sleep, ageusia, anosmia), was recorded from such diagnoses until death. These symptoms lasted several weeks or even months after infection, while some disappeared or recurred. This study was approved by the Cuerpo Médico Forense (Corte Suprema de Justicia de la Nación Argentina).

### 2.2. Tissue homogenates

*Frozen* tissues (0.5 cm^3^) were mechanically disaggregated using fine-point scissors and scalpel*s* in 0.6 mL of Dulbecco’s Modified Eagle Medium (DMEM). Homogenates were centrifuged at 12,000 × g for 10 min at 4°C, and supernatants were stored at −70°C until use.

### 2.3. Detection and quantification of SARS-CoV-2 genomic RNA

RNA was extracted using chemagic™ Viral DNA/RNA kit special H96 (PerkinElmer, Germany) on the automated chemagic™ 360 instrument (PerkinElmer, Germany). The efficiency of RNA extraction was independent of organ origin. RNA was quantified using a NanoDrop™ (Thermo Scientific™) and its load was normalized before SARS-CoV-2 RNA detection was performed using RT-qPCR (DisCoVery SARS-CoV-2 RT-PCR Detection Kit Rox) amplifying ORF1ab and N viral genes following the manufacturer’s instructions. The internal control was successfully amplified in all samples with a mean cycle threshold (Ct) value of 24.74 ± 3.19. Samples were defined as positive when Ct values were below 36 for both ORF1ab and N genes. The limit of detection (LOD) of this qualitative assay is 10 viral genomic RNA copies/mL.

Viral ORF1ab and N genomic RNA quantification were performed using a standard curve performed with quantified SARS-CoV-2 positive RNA control (GISAID EPI_ISL_420600) isolated from local viral strain cultured in Vero E6 cells and quantified against a WHO SARS-CoV-2 standard.

The viral load in samples was calculated by interpolation of the corresponding Ct value with a standard curve, which had been built with the Ct values obtained following PCR amplification of samples containing serial dilutions of quantified SARS-CoV-2 positive RNA control. The LOD for the quantitative assay is 100 SARS-CoV-2 genomic RNA copies/mL.

### 2.4. Amplification and sequencing of SARS-CoV-2 Spike gene

RT-PCR was done using a SuperScript III One-Step RT-PCR System with Platinum Taq High Fidelity DNA (Invitrogen). The pairs of primers used were the following: S-RBD Fw: 5´-CTTGTGCCCTTTTGGTGAAGT-3′ and Rv: 5´-GTGGATCACGGACAGCATCA-3′. The amplification cycle was 52°C for 30 min, 94°C for 2 min followed by 40 cycles: 94°C for 15 s, 58°C for 30s, 68°C for 90 s. Primer sets yielded a single product of the correct size.

The purified template DNA was bidirectionally sequenced using a BigDye Terminator Ready Reaction Cycle Sequencing Kit (Applied Biosystems) using the same primers pairs on an ABI Prism 3,130 Genetic Analyzer (Applied Biosystems). The obtained overlapping SARS-CoV-2 Spike sequences (nucleotide position 22,614 to 23,215 numbered according to GenBank accession number NC_045512) were then assembled using BLAST multiple sequences software by comparison with SARS-CoV-2 reference sequences. The sample information and corresponding GenBank accession number for each sample are listed in Table 1.

### 2.5. Sequence analysis

A distance-based phylogenetic tree based on S protein consensus sequences was constructed to infer the phylogenetic relationships between SARS-CoV-2 isolates obtained from each/tissue. Individual S gene nucleotide consensus sequences were first constructed using multiple sequence alignment *via* the ClustalW approach implemented in MEGA11. All ambiguous positions were removed for each sequence pair (pairwise deletion option). The evolutionary history among SARS-CoV-2 isolates from autopsy tissues and reference sequences from viral variants and sub-variants was inferred using the Neighbor-Joining method (Saitou and Nei, 1987). The distance-based neighbor-joining (NJ) phylogenetic tree was then constructed based on nucleotide sequences in MEGA 11 using bootstrap values of 1,000 replicates and a 70% threshold score (Tamura et al., 2021).

The tree is drawn to scale involving 69 nucleotide sequences, with branch lengths in the same units as those of the evolutionary distances used to infer the phylogenetic tree. The evolutionary distances were computed using the p-distance method (Nei, 2000) and are in the units of the number of base differences per site. The rate variation among sites was modeled with a gamma distribution (shape parameter = 1). Codon positions included were 1st + 2nd + 3rd + Noncoding. All ambiguous positions were removed for each sequence pair (pairwise deletion option). There were a total of 602 positions in the final dataset. Evolutionary analyses were conducted in MEGA11 (Tamura et al., 2021).

### 2.6. Cell culture and viral isolation from postmortem tissues

The African green monkey kidney cell line Vero E6 (ATCC, Rockville, MD) was cultured as monolayers in a 5% CO_2_ atmosphere at 37°C in DMEM (Sigma-Aldrich, Argentina) supplemented with 2 mM L-glutamine, 10% heat-inactivated fetal bovine serum (FBS) (Sigma-Aldrich, Argentina), 100 U/mL penicillin and 100 μg/mL streptomycin.

Vero E6 cells were seeded at a concentration of 8 × 10^4^ cells/well in 24-well plates and incubated with supernatants from frozen tissues that were mechanically disaggregated as described in point 2.2. The supernatants were diluted 1/4 in DMEM and incubated with the cells for 4 h at 37°C under a 5% CO_2_ atmosphere. The cells were then washed six times with DMEM, and the last wash was stored at −70°C to detect any remaining SARS-CoV-2 genomic RNA. The cells were incubated in DMEM supplemented with 2 mM L-glutamine, 2% heat-inactivated FBS, 100 U/mL penicillin, and 100 μg/mL streptomycin. Cytopathic effect, evidenced by the destruction of the cell monolayer observed under a light microscope at 20× magnification, was monitored every 24 h. After 7 days, the supernatants were inspected for the presence of SARS-CoV-2 RNA using RT-qPCR, as described in point 2.3.

Live SARS-CoV-2 manipulation was performed in biosafety level 3 facilities at the INBIRS.

## 3. Results

### 3.1. Detection and quantification of SARS-CoV-2 genomic RNA in tissues using RT-qPCR

Twenty-one cadavers referred to the Judicial Morgue of Argentina between January and August 2022 were studied. They involved 100 small tissue samples collected from five different organs (lung, heart, liver, kidney, and small intestine).

Twenty-one corpses sent to the Argentine Judicial Morgue between January and August 2022 were studied. From them, 100 small tissue samples were collected from five different organs: lung, heart, liver, kidney, and small intestine. The most frequent causes of death included adult respiratory distress syndrome with bilateral lung compromise during COVID-19 as well as exacerbations of preexisting comorbidities and COVID-19.

Qualitatively, SARS-CoV-2 RNA was found in all tissues analyzed for both viral target genes studied (ORF1ab, N), in 47 out of 100 tissue samples collected but showing dissimilar frequencies as follows: 13/21 in the lung, 7/21 in the heart, 9/21 in the liver, 8/21 in the kidney, as well as 10/16 in small intestine tissue.

Among the studied cases, the presence of SARS-CoV-2 RNA was verified in at least one tissue sample in all cases.

**Figure 1:**
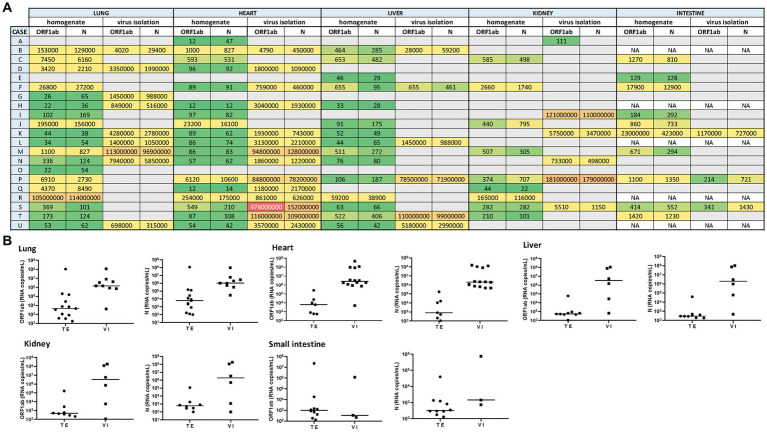
**(A)** Heat map representing the SARS-CoV-2 viral load recorded for each autopsy case from different tissues (including its homogenate and infectivity measurement in cell culture). A grid of colored squares ranging from green (minimum) to red (maximum) is used to represent the values of the viral load (expressed as copies/mL). The gray cells indicate an undetectable viral load, while “NA” indicates that the sample was not available. **(B)** The SARS-CoV-2 viral load (RNA copies/mL) was measured directly from tissue extracts (TE) and after 7-day-cell culture virus isolation (VI). The ORF1ab and N viral genes were detected in different tissues (lung, heart, liver, kidney, and small intestine) extract and culture supernatants from cell culture virus isolation.

Quantitatively, the highest viral load (expressed as median values in SARS-CoV-2 RNA copies/mL, for ORF1ab and N gene) was measured in lung samples (4.4 × 10^3^ and 6.2 × 10^3^). In this tissue, viral loads ranged from 1.01 × 10^2^ copies/mL to 1.14 × 10^8^ copies/mL. Comparatively, such median viral loads were lower in the liver (5.2 × 10^2^ and 2.9 × 10^2^), heart (6.1 × 10^3^ and 8.3 × 10^2^), kidney (4.7 × 10^2^ and 6.0 × 10^2^), and small intestine (9.8 × 10^2^ and 3.2 × 10^2^) (Figures 1A,B).

Overall, the median viral load measured in the tissue samples from non-hospitalized cases was lower than those hospitalized (1.4 × 10^3^ vs. 1.2 × 10^2^ copies/mL, respectively). The correlation was observed between the viral load and the levels of damage observed in the heart and lungs at the time of death.

### 3.2. SARS-CoV-2 Spike-based characterization and amino acid comparison according to tissue origin

**Figure 2:**
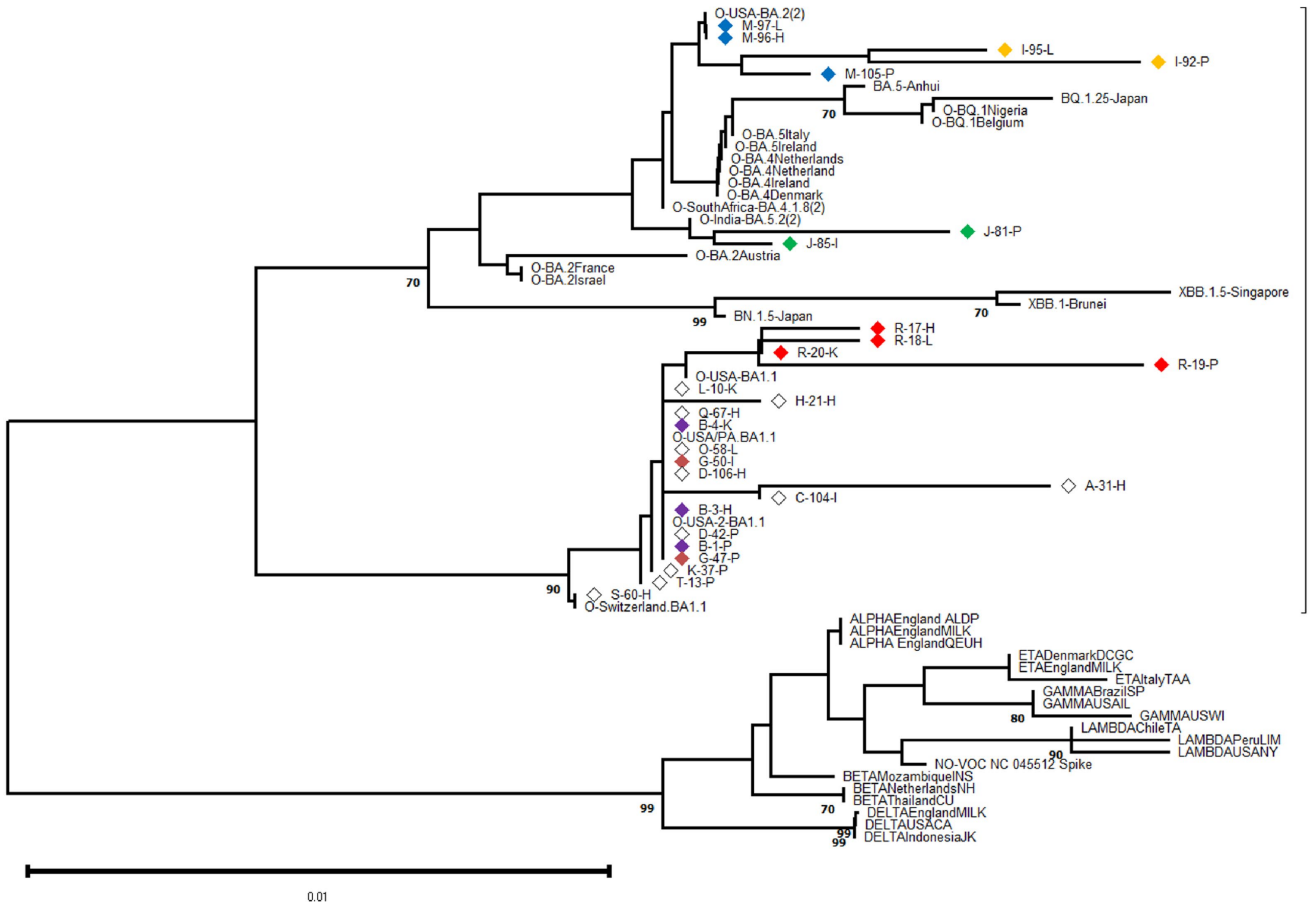
Phylogenetic analysis was performed on 602-base-pair-long nucleotide sequences of the SARS-CoV-2 Spike gene using the Neighbor Joining method with 1,000 bootstrap replicates. The lateral branches displayed the Omicron variant (as well as its sub-variants and recombinants represented by dashed lines), while the initially black branches represented the remaining non-Omicron variants. Each SARS-CoV-2 variant was accompanied by its reference sequence. The nucleotide sequences of SARS-CoV-2 isolates identified from autopsy materials were named according to the code provided in Table 1. Viral isolates from different tissues/cases were identified with a particular diamond color, while white diamonds represented unique viral isolates/cases. The numbers marked along the branches represented the bootstrap values higher than 0.7 out of 1,000 bootstrap resamplings. The tree scale depicted the number of substitutions/site.

Specimens (tissue samples) with a cycle-threshold (Ct) value of ≤25 in the SARS-CoV-2 RNA detection were considered appropriate for obtaining good-quality viral genome sequences (World Health Organization, 2022). Following this criterion and based on Spike-gene sequences, we were able to characterize 27 SARS-CoV-2 genomic RNA from 17 cases (excluding F, N, P, and U cases). The phylogenetic relatedness after NJ analysis showed that all S-gene nucleotide sequences clustered into the Omicron variant clade and were distributed into three distinguishable sub-variants, including 20 closely related to BA.1.1, two related to BA.5.2, and five sequences segregated in the BA.2 clade (Figure 2).

At the intra-host level, we characterized viral isolates from different tissues in six cases (B, G, I, J, M, R) at the genomic level. For each case, the viral isolates were closely related even when depicting minor Spike-gene nucleotide differences when comparing genomic RNA obtained from different tissues. The sequence analysis revealed that viral isolation from different tissues from one donor was highly related, revealing only minor Spike-gene nucleotide differences.

**Figure 3:**
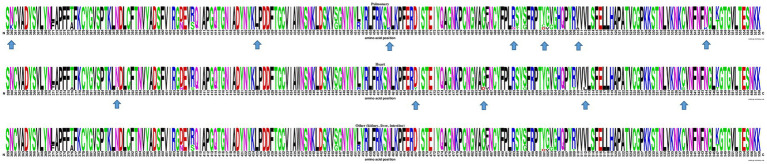
WebLogo profiles of SARS-CoV-2 receptor binding domain partial amino acid sequences (aa 359–558) characterized in viral isolates from different tissues are shown above. The upper profile represents sequences from the pulmonary tissue, the center profile represents sequences from the heart tissue, and the lower profile represents sequences from other tissues, including kidney, liver, and intestine. The size of the letter (bits; *Y* axis) indicates the frequency of the amino acid substitutions at a certain residue position (*X* axis). Different residues at the same position are scaled according to their frequency and colored based on their amino acid characteristics. An arrow is used to indicate RBD amino acid positions with a distinctive but infrequent residue.

At the inter-host level, there was no clustering among S-gene sequences according to any particular tissue of origin (Figure 2). Similarly, when comparing the Spike amino acid sequences (particularly in the Receptor Binding Domain or RBD) obtained from pulmonary tissues (*n* = 9), heart (*n* = 8), and other organs including kidney, liver, and intestine (*n* = 10), minor contributions in the frequency of amino acids were observed (Figure 3).

### 3.3. Recovery of infectious SARS-CoV-2 using cell culture isolation

Virus isolation from the five tissue samples that were studied (lung, heart, liver, kidney, and small intestine) was performed using supernatants from disaggregated tissues. After 7 days, the presence of infectious SARS-CoV-2 was evidenced by the cytopathic effect on Vero E6 monolayers.

Thus, infectious SARS-CoV-2 was confirmed after supernatants from tissue extract added on Vero E6 cells in 9 out of 21 lung samples (2 from reinfection cases), as well as in extrapulmonary organs, including 14 out of 21 hearts (4 from reinfection cases), 6 out of 21 livers (3 from reinfection cases), 6 out of 21 kidneys (1 from reinfection cases), and 3 out of 16 small intestine samples (1 from reinfection cases) (Figures 1A,B).

After 7 days, to determine the presence of infectious (that is, replication-competent) SARS-CoV-2 in tissue specimens, we carried out its recovery in a Vero cell monolayer. We have assessed the plaque formation as the lytic cytopathic effect. Thus, infectious SARS-CoV-2 was confirmed after supernatants from tissue extract added on Vero E6 cells identified in 9 out of 21 lung extracts samples (3 from reinfection cases), as well as in extrapulmonary organs, including 14 out of 21 hearts (5 from reinfection cases), 6 out of 21 livers (2 from reinfection cases), 6 out of 21 kidneys (2 from reinfection cases), and 3 out of 16 small intestine samples (2 from reinfection cases) (Figures 1A,B).

Tissue samples from 6 out of the 8 COVID-19 vaccine recipients were included among those with confirmed viral infectivity. Moreover, infectious virus was detected in tissue samples from 5 out of 9 lungs, 6 out of 14 hearts, 2 out of 6 livers, and 3 out of 6 kidneys, in which genomic RNA was undetectable upon direct analysis. Because no viable virus or viral RNA was detected in the cell culture media used for the last wash, it can be concluded that the isolated virus came from viral replication.

The highest viral load measured by RT-qPCR in 7-day/cell culture supernatants (expressed as median values in copies/mL, for ORF1ab and N gene) were found in the lung (1.4 × 10^6^ and 1.06 × 10^6^) and heart (1.9 × 10^6^ and 1.6 × 10^6^) samples. Such viral load decreased slightly in the liver (3.3 × 10^6^ and 2 × 10^6^), small intestine (3.4 × 10^2^ and 1.4 × 10^3^), and kidney (3.2 × 10^6^ and 2 × 10^6^) samples.

## 4. Discussion

Autopsy material originating from those with an antemortem COVID-19 diagnosis is contagious thanks to the presence of SARS-CoV-2. Based on previous autopsy series (Desai et al., 2020; Trypsteen et al., 2020; Vincent and Taccone, 2020; Wolfel et al., 2020; Damiani et al., 2021), SARS-CoV-2 primarily infects the lungs and then disseminates through the blood, infecting cells of the different permissive organs and tissues. Several studies report the persistence of viral RNA shedding for an extended period after the onset of acute symptoms, as well as the presence of viral RNA and/or antigen in the gastrointestinal tissues of convalescent patients (Lamers et al., 2020; Trypsteen et al., 2020; Martin-Cardona et al., 2021; Cheung et al., 2022; Zollner et al., 2022).

The presence of replicating SARS-CoV-2 in samples from cadavers has been previously suggested by the detection of subgenomic SARS-CoV-2 RNA in post-mortem nasopharyngeal swabs (Schroder et al., 2021; Grassi et al., 2022). However, this finding is limited by the fact that the detection of SARS-CoV-2 subgenomic RNAs is not a definitive indicator of active replication (Alexandersen et al., 2020). The presence of viable SARS-CoV-2 in different autopsy tissues was recently reported (Plenzig et al., 2021a; Stein et al., 2022) but it was not possible to observe the same in samples obtained from two exhumed corpses (Plenzig et al., 2021b).

This study includes 21 cases (10 of them with previous hospitalization) with post-mortem nasopharyngeal swabs that tested positive for the presence of SARS-CoV-2 RNA. The autopsies were carried out from which 100 tissue samples were collected including lung, heart, liver, kidney, and small intestine.

We have demonstrated the presence of SARS-CoV-2 genomic RNA, as well as the presence of the replication-competent virus, providing certain proof of the viability of the virus regardless of COVID-19 vaccination status. However, we do not have data available on the presence of neutralizing antibodies against the virus. Five cases of SARS-CoV-2 reinfection were found, and considering the clinical records they presented several clinical signs and symptoms compatible with post-acute COVID. Importantly, viral particles were isolated from tissue samples from these cases, and the genomic characterization of SARS-CoV-2 did not offer any evidence of prevalent viral variants, such as Delta when their first infection diagnosis was carried out, thus offering additional support for the reinfection event.

Because SARS-CoV-2 has numerous distinctive mutations, it can be recognized from partial S-gene direct sequences, including the RBD (European Centre for Disease Prevention and Control, 2021; World Health Organization, 2021). Therefore, we used this approach to detect the presence of three different Omicron sub-variants, including BA.1.1 (20 out of 27 isolates), BA.2 (5 out of 27), and BA.5.2 (2 out of 27). Such distribution matches the Argentinean epidemiology map during the sampling time (Torres et al., 2023). However, further research is warranted, as whole-genome sequencing (WGS) of SARS-CoV-2 using next-generation sequencing (NGS) technology remains the gold standard for tracking and identifying new variants, especially for characterizing minority variants, including preexistent ones (Berno et al., 2022).

Among the studied cases, there were COVID-19 vaccine recipients with completed or unscheduled vaccinations, and even with hybrid immunity. Breakthrough infections with Omicron sub-variants are frequent, despite the strong protection provided by COVID-19 mRNA vaccines and even hybrid immunity against SARS-CoV-2 variants of concern (Van Zelm, 2022; Pradenas et al., 2023).

Interestingly, the tissues studied revealed the presence of SARS-CoV-2 by detecting its genomic RNA and/or its infectivity in cell culture. Thus, autopsies of people who have died from COVID-19 are associated with a reliable biological risk because the virus remains viable in several organs. Although the quantities were widely varied in most cases, viral loads in lung tissue samples appeared higher than in other organs, which probably implies host-related undetermined factors, such as increased affinity to the receptor of human cells and less efficient viral clearance between different tissues (Beyer and Forero, 2022). Likewise, the antemortem clinical condition was diverse among the cases studied, suggesting that the SARS-CoV-2 viral load in lung may vary greatly according to the proliferative and exudative phases of diffuse alveolar damage, which itself drives the multiple organ dissemination of SARS-CoV-2 (Delorey et al., 2021; Odilov et al., 2021). Because tissue samples were collected within a short postmortem interval (a mean of 1.9 days), even between hospitalized and non-hospitalized cases, we have minimized the bias due to cellular autolysis, associated with postmortem changes that alter the extracellular space by releasing intracellular content and influencing the postmortem viral load. Nevertheless, other tissue and cell-related factors may be involved such as their differential cellular permissiveness and the relative abundance of super-permissive cells (Lee et al., 2022). Furthermore, the absence of SARS-CoV-2 RNA tested in tissue homogenate did not necessarily correlate with the absence of viable SARS-CoV-2. Additionally, the presence of viral RNA did not necessarily implicate the presence of infective viral particles.

It could reflect the persistence of RNA in tissues even when the infectious virus has been eliminated (Griffin, 2022). Lung, heart, and kidney samples revealed their infectiousness only when SARS-CoV-2 was isolated in cell culture.

The infectivity time for the SARS-CoV-2 in fresh cadaveric tissue and exhumed corpses even after months in an earth grave warrants further investigation. Furthermore, as the number of individuals recovered from COVID-19 is increasing, it is mandatory to define whether and when organs from these potential donors can be safely used. Few available reports among donors with prior COVID-19 infection indicate low rates of transmission to the recipient and transplanted organ dysfunction, depending on the infection status (Kute et al., 2021; Saharia et al., 2023). However, few studies have been conducted until now, indicating that viral transmission by organ donation could not be completely ruled out.

In conclusion, this study reinforces that autopsies of COVID-19 cases are associated with a consistent biological risk because the virus remains viable and with a high load, necessitating utmost caution in the handling of all corpses.

Thus, the studies involving potentially infected corpses should be performed in adequate biosafety facilities only by expert professionals and technical operators wearing the correct protective equipment following the international biosafety normative (Centers for Disease Control and Prevention, 2020).

Negative clinical records of SARS-CoV-2 do not guarantee microbiologically negative yields, highlighting the importance of carrying out additional clinical tests and examinations in organ donors who have recovered from SARS-CoV-2 infection. In the end, this research makes a strong argument for continuing research of this kind in the future to characterize the mechanisms underlying post-acute COVID-19 sequelae in the pulmonary and wide range of extrapulmonary organ systems. It also adds to our understanding of the virus’s multiple tissue tropisms even after reinfection and plausible persistence of SARS-CoV-2.

## Data availability statement

The datasets presented in this study can be found in online repositories. The names of the repository/repositories and accession number(s) can be found in the article/supplementary material.

## Author contributions

MD and JQ contributed equally to the conception and design of this study, funding acquisition, project administration, acquisition, analysis, interpretation of data, and writing of the manuscript. SM-B, AD’A, and CB were responsible for the autopsy and sample collection at Morgue. CC focused on bioinformatics analysis of data. FR SARS-CoV-2 viral load data curation. RF, CL, AG, FS, CC, and PJ contributed to molecular biology experiments. All authors contributed to the article and approved the submitted version.

## Funding

This study was supported by the Argentinean National Agency of Scientific and Technological Promotion (grant number PICTO 2021-00005 to JQ). The funder had no role in the study design, data collection, interpretation, or the decision to submit the work for publication.

## Conflict of interest

The authors declare that the research was conducted in the absence of any commercial or financial relationships that could be construed as a potential conflict of interest.

## Publisher’s note

All claims expressed in this article are solely those of the authors and do not necessarily represent those of their affiliated organizations, or those of the publisher, the editors and the reviewers. Any product that may be evaluated in this article, or claim that may be made by its manufacturer, is not guaranteed or endorsed by the publisher.
